# Systematic Pathway Enrichment Analysis of a Genome-Wide Association Study on Breast Cancer Survival Reveals an Influence of Genes Involved in Cell Adhesion and Calcium Signaling on the Patients’ Clinical Outcome

**DOI:** 10.1371/journal.pone.0098229

**Published:** 2014-06-02

**Authors:** Andrea Woltmann, Bowang Chen, Jesús Lascorz, Robert Johansson, Jorunn E. Eyfjörd, Ute Hamann, Jonas Manjer, Kerstin Enquist-Olsson, Roger Henriksson, Stefan Herms, Per Hoffmann, Kari Hemminki, Per Lenner, Asta Försti

**Affiliations:** 1 Division of Molecular Genetic Epidemiology, German Cancer Research Center (DKFZ), Heidelberg, Germany; 2 Department of Radiation Sciences & Oncology, Umeå University, Umeå, Sweden; 3 Cancer Research Laboratory, Faculty of Medicine, University of Iceland, Reykjavik, Iceland; 4 Molecular Genetics of Breast Cancer, German Cancer Research Center (DKFZ), Heidelberg, Germany; 5 The Malmö Diet and Cancer Study, Lund University, Malmö, Sweden; 6 Department of Plastic Surgery, Skåne University Hospital, Malmö, Lund University, Malmö, Sweden; 7 Department of Public Health and Clinical Medicine/Nutritional, Research, Umeå University, Umeå, Sweden; 8 Cancer Center Stockholm Gotland, Stockholm, Sweden; 9 Institute of Human Genetics, Department of Genomics, University of Bonn, Bonn, Germany; 10 Division of Medical Genetics and Department of Biomedicine, University of Basel, Basel, Switzerland; 11 Center for Primary Health Care Research, Clinical Research Center, Lund University, Malmö, Sweden; Institute of Molecular and Cell Biology, Biopolis, United States of America

## Abstract

Genome-wide association studies (GWASs) may help to understand the effects of genetic polymorphisms on breast cancer (BC) progression and survival. However, they give only a focused view, which cannot capture the tremendous complexity of this disease. Therefore, we investigated data from a previously conducted GWAS on BC survival for enriched pathways by different enrichment analysis tools using the two main annotation databases Gene Ontology (GO) and Kyoto Encyclopedia of Genes and Genomes (KEGG). The goal was to identify the functional categories (GO terms and KEGG pathways) that are consistently overrepresented in a statistically significant way in the list of genes generated from the single nucleotide polymorphism (SNP) data. The SNPs with allelic *p*-value cut-offs 0.005 and 0.01 were annotated to the genes by excluding or including a 20 kb up-and down-stream sequence of the genes and analyzed by six different tools. We identified eleven consistently enriched categories, the most significant ones relating to cell adhesion and calcium ion binding. Moreover, we investigated the similarity between our GWAS and the enrichment analyses of twelve published gene expression signatures for breast cancer prognosis. Five of them were commonly used and commercially available, five were based on different aspects of metastasis formation and two were developed from meta-analyses of published prognostic signatures. This comparison revealed similarities between our GWAS data and the general and the specific brain metastasis gene signatures as well as the Oncotype DX signature. As metastasis formation is a strong indicator of a patient’s prognosis, this result reflects the survival aspect of the conducted GWAS and supports cell adhesion and calcium signaling as important pathways in cancer progression.

## Introduction

Worldwide, breast cancer (BC) is the most common cancer among women, comprising 23% of all female cancer. Each year, about 1.4 million new cases are diagnosed and about 460,000 women die of this disease [1]. It has been shown that survival of BC is in part heritable which can possibly be explained by yet unknown genetic factors [2]. Further knowledge about the effects of inherited genetic variation on BC survival can help to predict the patient’s individual risk for disease progression and survival probabilities and to develop new and better therapies and prevention strategies. A genome-wide association study (GWAS) is a powerful tool to search for a genetic influence on complex traits. Within the last six years 34 GWASs on breast cancer have been performed identifying 194 new susceptibility loci (http://www.genome.gov/gwastudies). Also three GWASs on breast cancer survival have been conducted leading only to three prognostic loci [3]–[5]. Therefore, a more global view on GWAS data can reveal new insights in cancer formation and progression and give new clues for further investigations.

A good tool to set high-throughput data into a global context is a pathway enrichment analysis [6]. The gene-group-based approach increases the likelihood to identify the biological processes which are overrepresented in the high-throughput data and have a high impact on the studied disease. The most commonly used gene annotation databases are Gene Ontology (GO) and Kyoto Encyclopedia of Genes and Genomes (KEGG), in which the biological knowledge about genes and their associated processes and pathways are collected. This knowledge can be used by pathway enrichment tools, which map the genes of the investigated list to the associated biological annotation terms of the database. Then, the customized enrichment result is compared to the control background and an enrichment *p*-value is calculated and corrected for multiple testing. Currently, a huge variety of different pathway enrichment tools are available.

Some of the tools input lists of genes or proteins and output enriched pathways. Others take the locations of single nucleotide polymorphisms (SNPs) into consideration, and thus gene lists can be derived from GWAS data. The aim of our study was to submit a GWAS on BC survival to a pathway enrichment analysis. In the GWAS, the genotype data of women of Western European origin with long and short time survival after the diagnosis of BC were compared. Pathway enrichment analysis was conducted using six different enrichment tools on four final gene lists. The gene lists based on our SNP data with allelic *p*-value cut-offs 0.005 and 0.01 and with a gene annotation by excluding or including a 20 kb up- and down-stream sequence of the gene. Only those categories which were enriched in all four lists and more than one tool were considered to be consistently enriched. We were also interested whether our results are supported by gene signatures on breast cancer prognosis derived from gene expression profiling studies. Therefore, we performed pathway enrichment analyses with several commonly used prognostic gene signatures and compared the results with our GWAS data.

## Materials and Methods

### Ethics Statement

All participants in the GWAS gave written informed consent to the use of their samples for research purpose. The study was approved by the ethical committee of each participating institute.

### GWAS

The GWAS on BC survival was a population based case-only study, in which the BC patients were divided in two groups based on their survival time. We considered as cases 369 women with short-time survival (less than 6 years after breast cancer diagnosis) which were compared with a group consisting of 369 women with long-time survival (≥11 years after breast cancer diagnosis) as controls. Details of the characteristics of the study population are found in the table S1. The cases and controls were selected from four cohorts and matched for age at diagnosis (<40, 40–49, 50–59 and ≥60 years), gender, diagnosis period (1985–1989, 1990–1994 and 1995–) and cohort (table S2). Blood samples were prospectively collected in each cohort. The cases and controls were identified from the cohorts by record linkage to the regional cancer registries. Follow-up was performed until December, 31st, 2007 and the data were available for every patient. The Västerbotten intervention project (VIP), the mammary screening project (MSP) and the Department of Oncology, Norrlands University Hospital, Umeå, Sweden, contributed with 96 cases and 96 controls [7]. Within VIP, blood samples have been collected since 1985, within MSP since 1995, with subsequent BC diagnosis during the years 1988–2005. Norrlands University Hospital collects blood samples consecutively since 1990 from newly diagnosed BC patients and 43 BC patients, not included in VIP or MSP, were included in the study. The Malmö Diet and Cancer Study, Malmö, Sweden contributed 44 cases and 44 controls [8], [9]. Blood samples were collected between 1991 and 1996, prior to BC diagnosis between 1991 and 2005. The third sample set comprised 82 cases and 14 controls from the Städtisches Klinikum Karlsruhe and Deutsches Krebsforschungszentrum Breast Cancer Study (SKKDKFZS) and 68 controls from the Umeå cohort. The SKKDKFZS consists of women between 21–93 years of age at diagnosis with pathologically confirmed breast cancer recruited at the Städtisches Klinikum Karlruhe, Karlsruhe, Germany from 1993–2005 and a blood sample collected at the time of diagnosis. The Icelandic Cancer Society and University of Iceland Biobank contributed with 147 cases and 147 controls with BC diagnosis during the years 1983–2004 [10].

A genome-wide scan of ∼ 300,000 tagging SNPs was conducted using the Illumina HumanCytoSNP-12 v1 according to the manufacturer’s protocols. Before analysis, markers with one or more of the following criteria were excluded: <90% genotype call rate, minor allele frequency <5% or Hardy–Weinberg equilibrium exact *p*-value <10^−5^. Genotype calling was done using Illumina GenomeStudio 2010. The GWAS was conducted by PLINK v1.06, with the option of “model” to perform a Cochran-Armitage and a full-model case-control association test.

### Enrichment Analysis

The GWAS data were investigated for SNPs which were annotated to a gene and located within the 5′UTR, 3′UTR, introns and exons of the gene, alternatively within a genomic region including up to 20 kb up- and downstream of a gene locus. Different allelic *p*-value cut-offs (0.05, 0.01, 0.005, 0.001 and 0.0001) were set to generate gene lists for both scenarios. If there was more than one SNP per gene meeting the selection criteria, the SNP with the lowest *p*-value was taken into account. Finally, four gene lists, two per scenario, with the allelic *p*-value cut offs of 0.01 and 0.005 were selected as input for six enrichment analysis tools (ConsensusPathDB, DAVID, FatiGO, GATHER, GeneCodis and WebGestalt) using the two main annotation databases Gene Ontology (GO) and Kyoto Encyclopedia of Genes and Genomes (KEGG) as basis. These pathway enrichment tools were selected based on our previous experience on pathway enrichment analyses [11]. The selection criteria included free availability, user-friendly handling, the usage of gene names as input and the GO and the KEGG database as basis, variations in stringency, test statistics and multiple comparison adjustment methods. The four gene lists were selected because they provided the enrichment tools with an applicable number of genes to run the enrichment analyses successfully. With a too stringent allelic SNP *p*-value cut-off, too few genes serve as input resulting in no significantly enriched categories. A too tolerant allelic *p*-value cut-off increases the background noise and may result either in too many unspecific or no enriched categories [12], [13]. For all tools the same conditions were applied, which were a significance threshold of 0.05 for the adjusted enrichment *p*-value, at least two genes from the input list in the enriched category and the whole genome as reference background. The goal was to identify the functional categories (GO terms and KEGG pathways) that are consistently overrepresented in a statistically significant way in the list of SNPs inferred from the GWAS on BC prognosis. The used tools and their characteristics can be seen in table S3.

### Consistently Enriched Categories

**Figure 1:**
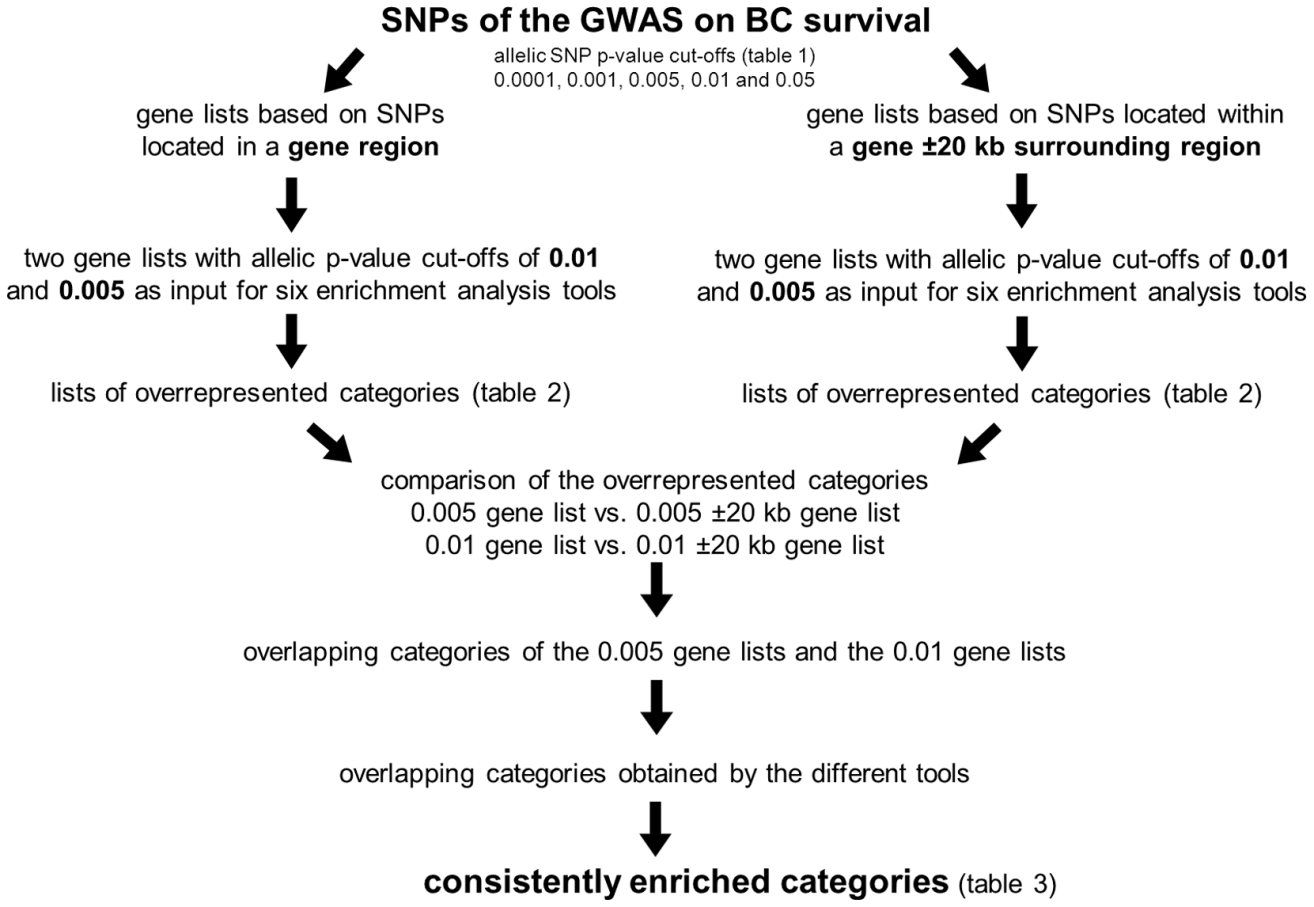
Flow chart of the pathway enrichment analysis of the GWAS on BC survival.

For DAVID, FatiGO and GATHER, the tool’s default *p*-value cut-off of 0.05 generated a list of 20–30 enriched categories for the comparison. However, for Consensus PathDB, GeneCodis and WebGestalt, this *p*-value cut-off generated lists of up to 176, 278 and 77 enriched GO terms and we used the more stringent enrichment *p*-value cut-offs of 1×10^−6^, 1×10^−4^ and 0.01, respectively. The enriched categories of each allelic *p*-value cut-off gene list based on the SNPs within a gene region were compared to those of the gene list taking also the SNPs within the ±20 kb spanning region into account (0.01 list vs. 0.01±20 kb list; 0.005 list vs. 0.005±20 kb list). This was done for every tool separately. Then, the overlaps of the two different allelic *p*-value cut-offs were compared to each other. Finally, we compared the results of all tools to each other. Only categories enriched in all four gene lists and by more than one tool were considered consistently enriched (figure 1).

### Prognostic Gene Expression Signatures

Literature was searched for commonly used prognostic gene signatures derived from breast cancer expression data. Twelve gene expression signatures were selected for further pathway enrichment analysis conducted by MetaCore GeneGo pathway enrichment analysis (false discovery rate (FDR) cut-off 0.05) because this tool enables the pathway enrichment analysis of two gene lists simultaneously and compares the results to each other. Also the 0.01 gene list of our GWAS was analyzed again with this tool to make the results comparable to the ones of the gene expression signatures.

## Results

### Systematic Enrichment Analyses of the GWAS Data

The consecutive steps of the pathway enrichment analysis are summarized in figure 1. The GWAS data were filtered for SNPs located within a gene (5′UTR, 3′UTR, intron and exon), as well as for SNPs located in a genomic region 20 kb up- and downstream from a gene locus to take also genetic effects in regulatory regions into account. Five different *p*-value cut-offs for both scenarios were set to generate gene lists (table 1). The gene lists based on SNPs created by the *p*-value cut-offs 0.01 and 0.005, consisting of 737 and 1143 genes and 402 and 638 genes, respectively, provided the enrichment tools with an applicable number of genes to run the enrichment analyses successfully.

**Table 1:** Number of SNPs and genes corresponding to allelic *p*-value cut-offs of the GWAS on BC survival.

*p*-value	total No. of SNPs	No. of SNPs within a gene	No. of genes	No. of SNPs ±20 kb	No. of genes ±20 kb
<0.05	4572	1664	1015	2525	1576
<0.01	3080	1137	737	1725	1143
<0.005	1607	576	402	746	638
<0.001	329	112	83	163	125
<0.0001	40	9	9	12	10

These four gene lists served as input in six different pathway enrichment analysis tools under the same setting. The number of enriched GO terms/KEGG pathways differed enormously between the enrichment analysis tools due to the individual tool features although the same analysis conditions were assigned (table 2). The ConsensusPathDB and GeneCodis tool reported in general much more enriched GO terms than the other used tools. For example, they generated 176 and 278 overrepresented GO annotations, respectively, when the 0.01±20 kb gene list was analyzed. As comparison, DAVID and FatiGO reported only 4 enriched categories each.

**Table 2:** Number of GO annotations and KEGG pathways enriched by six pathway enrichment tools for gene lists with allelic SNP *p*-value cut-offs 0.005 and 0.01.

	SNP *p*-value cut-off 0.005				SNP *p*-value cut-off 0.01			
	within a gene		gene locus ±20 kb		within a gene		gene locus ±20 kb	
Tool name	No. of GO Annotations	No. of KEGG pathways	No. of GO Annotations	No. of KEGG pathways	No. of GO Annotations	No. of KEGG pathways	No. of GO Annotations	No. of KEGG pathways
ConsensusPathDB	159	16	170	14	161	25	176	21
DAVID	32	0	15	0	22	5	4	1
FatiGO	7	0	5	0	5	0	4	0
GATHER	20	3	10	2	33	5	23	4
GeneCodis	133	11	172	15	223	29	278	26
WebGestalt	50	15	24	17	77	37	52	37

Pathway enrichment *p-*value cut-off: 0.05.

To reduce the number of enriched categories, the results of the two gene lists with an allelic *p*-value cut-off of 0.005 were compared with each other. This was also done for the two gene lists with a *p*-value cut-off of 0.01 and the resulting overlaps were compared with each other. This was done separately for every tool. To define consistently enriched categories, the categories had to be overrepresented by at least two different tools. After this comparison eleven categories remained: two GO terms, which were “calcium ion binding” and “cell adhesion” and nine KEGG pathways named “adherens junction”, “arrythmogenic right ventricular cardiomyopathy”, “axon guidance”, “calcium signaling”, “dilated cardiomyopathy”, “ECM-receptor interaction”, “focal adhesion”, “O-glycan-biosynthesis” and “small cell lung cancer” (table 3). Most categories were reported by three or four tools.

**Table 3:** Consistently enriched categories of the GWAS on BC survival.

GO Annotations (6 tools)		Number of tools	Number of genes in category	Number of GWAS genes* in category
GO:0005509	calcium ion binding	3	685	67
GO:0007155	cell adhesion	4	958	52
**KEGG Pathways (4 tools)**				
KEGG 04520	Adherens junction	3	59	9
KEGG 05412	Arrhythmogenic right ventricular cardiomyopathy	2	65	10
KEGG 04360	Axon guidance	3	80	15
KEGG 04020	Calcium signaling pathway	3	139	17
KEGG 05414	Dilated cardiomyopathy	3	82	11
KEGG 04512	ECM-receptor interaction	4	57	8
KEGG 04510	Focal adhesion	3	134	15
KEGG 00512	O-Glycan biosynthesis	2	11	7
KEGG 05222	Small cell lung cancer	2	65	6

Only categories enriched in all four gene lists and by more than one tool were considered consistently enriched. * Genes present in the 0.01 gene list (allelic SNP *p*-value cut-off 0.01).

We compared the genes of every category to each other to detect overlaps of the pathways to define the consistently enriched categories (table 4). The cross-tabulation revealed a strong association of “cell adhesion” genes with all pathways except for the genes in “calcium signaling” and “O-glycan biosynthesis”. Moreover, we investigated the overlap of our GWAS genes in the pathways, resulting in a similar outcome (table 5). Based on this analysis most categories were summarized in two overarching categories:

**Table 4:** Gene overlap of the consistently enriched categories for all pathway genes.

				a	b	c	d	e	f	g	h	i	j	k
	ID	Category	Number of genes	685	958	59	65	80	139	82	57	134	11	65
**a**	GO:0005509	Calcium ion binding	685	–	185	0	6	3	14	8	1	9	0	0
**b**	GO:0007155	Cell adhesion	958		–	21	29	25	5	25	48	63	0	23
**c**	KEGG 04520	Adherens junction	59			–	8	9	2	1	0	17	0	0
**d**	KEGG 05412	Arrhythmogenic right ventricular cardiomyopathy	65				–	1	5	51	21	23	0	6
**e**	KEGG 04360	Axon guidance	80					–	2	1	1	20	0	2
**f**	KEGG 04020	Calcium signaling pathway	139						–	18	0	6	0	1
**g**	KEGG 05414	Dilated cardiomyopathy	82							–	21	23	0	6
**h**	KEGG 04512	ECM-receptor interaction	57								–	42	0	18
**i**	KEGG 04510	Focal adhesion	134									–	0	32
**j**	KEGG 00512	O-Glycan biosynthesis	11										–	0
**k**	KEGG 05222	Small cell lung cancer	65											–

**Table 5:** Gene overlap of the consistently enriched categories based on the GWAS genes present in the 0.01 gene list.

				a	b	c	d	e	f	g	h	i	j	k
	ID	Category	Number of genes	67	52	9	10	15	17	11	8	15	7	6
**a**	GO:0005509	Calcium ion binding	67	–	13	1	8	1	11	7	2	4	6	0
**b**	GO:0007155	Cell adhesion	52		–	3	5	3	0	3	7	10	0	5
**c**	KEGG 04520	Adherens junction	9			–	2	1	0	0	0	2	0	0
**d**	KEGG 05412	Arrhythmogenic right ventricular cardiomyopathy	10				–	1	2	8	3	4	0	1
**e**	KEGG 04360	Axon guidance	15					–	1	1	1	2	0	1
**f**	KEGG 04020	Calcium signaling pathway	17						–	4	0	1	0	0
**g**	KEGG 05414	Dilated cardiomyopathy	11							–	3	3	0	1
**h**	KEGG 04512	ECM-receptor interaction	8								–	7	0	5
**i**	KEGG 04510	Focal adhesion	15									–	0	5
**j**	KEGG 00512	O-Glycan biosynthesis	7										–	0
**k**	KEGG 05222	Small cell lung cancer	6											–

1. “Cell adhesion” with its 52 GWAS genes combined different kinds of cell adhesion processes, such as the KEGG pathways “adherens junction”, “ECM-receptor interaction”, “focal adhesion”, as well as “small cell lung cancer” and to a lesser extent “axon guidance”.
2. “Calcium ion binding” characterizes the group of KEGG pathways “arrythmogenic right ventricular cardiomyopathy”, “calcium signaling”, “dilated cardiomyopathy” and “O-glycan biosynthesis”.

Additionally, the gene overlap of 20–30% between the two GO terms “calcium ion binding” and “cell adhesion” supports a connection of these two annotations.

### Enrichment Analysis of the 0.01 Gene List with MetaCore

**Figure 2:**
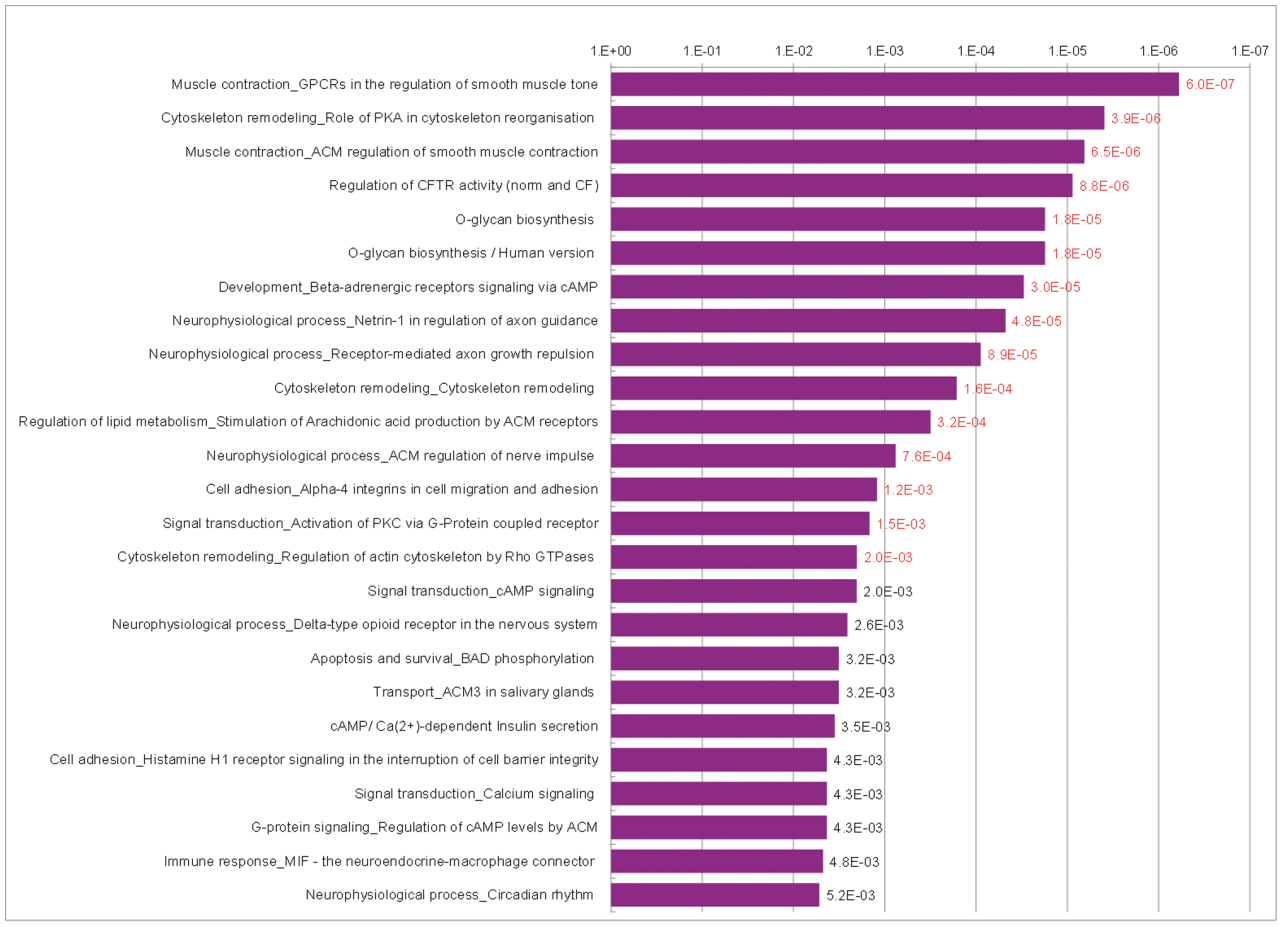
Top 25 GeneGO pathways enriched by the 0.01 gene list derived from the GWAS data. red numbers = significant at FDR of 0.05.

As we wanted to compare our GWAS pathways with prognostic expression signatures, the longer 0.01 gene list was further analyzed by GeneGo, the pathway enrichment analysis tool of MetaCore, which allows simultaneous analysis and comparison of two gene lists. Fifteen pathways passed the significance level defined by a FDR of 0.05 and the analysis confirmed the importance of cell adhesion, axon guidance and calcium signaling (figure 2) in the GWAS survival signature. Although the GeneGo pathway enrichment analysis uses its own pathway terms, they are similar to the GO terms or KEGG pathways. Also the O-Glycan biosynthesis was found in the top 5 enriched pathways. The five most common terms, cell adhesion, cytoskeleton remodeling, development, muscle contraction and neurophysiological process, constituted 56% of the top 50 pathways (table 6, table S4).

**Table 6:** Distribution of the seven generic terms among the 50 top pathways in the enrichment analyses of the 13 gene lists.

	signature name	No. ofgenes	cytoskeletonremodeling		celladhesion		cellcycle		neurophysio- logicalprocess		musclecontraction		immuneresponse		development		sum ofpathways	
			total	%	total	%	total	%	total	%	total	%	total	%	total	%	total	%
**based on** **GWAS data**	0.01 gene list	737	6	12	6	12	0	0	6	12	3	6	1	2	7	14	29	58
**commercially used** **gene signatures**	Mammaprint	70	1	2	3	6	3	6	5	10	2	4	3	6	12	24	29	58
	Oncotype DX	21	5	10	4	8	9	18	1	2	0	0	1	2	10	20	30	60
	MapQuant	97	4	8	1	2	17	34	0	0	0	0	2	4	1	2	25	50
	Gene Search	76	2	4	3	6	9	18	2	4	0	0	6	12	6	12	28	56
	Wound responsesignature	512	5	10	3	6	3	6	0	0	0	0	4	8	4	8	19	38
**special metastasis** **gene signatures**	Lung metastasissignature	54	4	8	5	10	0	0	0	0	0	0	16	32	11	22	36	72
	Brain metastasissignature	243	2	4	6	12	0	0	0	0	1	2	18	36	11	22	38	76
	Bone metastasissignature	102	6	12	4	8	0	0	0	0	0	0	14	28	11	22	35	70
**general metastasis** **gene signatures**	Invasivenesssignature	186	4	8	1	2	1	2	1	2	0	0	8	16	7	14	22	44
	Generalmetastasissignature	128	4	8	4	8	8	16	2	4	3	6	4	8	5	10	30	60
**derived from** **meta-analyses**	Meta genesignature	376	0	0	0	0	17	34	0	0	0	0	5	10	3	6	25	50
	374 GeneSet/consensusgenes	374	5	10	3	6	17	34	0	0	0	0	3	6	3	6	31	62

### Enrichment Analyses of the Gene Expression Signatures

Literature was searched for commonly used prognostic gene signatures derived from breast cancer expression data. We selected twelve signatures for further pathway enrichment analysis (table 7). Mammaprint [14], Oncotype DX [15], MapQuant [16], Gene Search [17] and the fibroblast core serum response (CSR) signature, commonly known as wound response signature [18], are well established, often cited in literature and commercially available. We also included five gene signatures based on expression data of metastatic breast cancer or metastatic adenocarcinomas of diverse origin [19]–[23], because metastasis formation has a profound impact on patients’ survival. Last, we added two prognostic gene signatures based on meta-analyses of published gene expression signatures and microarray data sets of breast tumors [24], [25] to evaluate how a combination of several prognostic gene signatures influences the enrichment analysis and if this result is comparable to the one obtained by the GWAS.

**Table 7:** Prognostic gene expression signatures selected for pathway enrichment analysis.

Signaturename	Author	Year ofpublication	No. ofgenes	Study design	Outcome
Mammaprint	Van’t Veer*et al.* [14]	2002	70	78 patients with sporadic primary breast tumors:<5 cm, N0, age <55 years; 34 patients developeddistant metastasis <5 years vs. 44 patients:disease-free >5 years	Prognosis for distantmetastasis
Oncotype DX	Paik *et al.* [15]	2004	21	668 tumors from patients: N0 and ER+,treated with tamoxifen	Prognosis for distantrecurrence/overall survival
MapQuant	Sotirou *et al.* [16]	2006	97	64 samples: ER+, grade 1 vs. grade 3	Prognosis for recurrence/relapse-free survival
Gene Search	Wang et al.[17]	2005	76	115 tumors: all N0; 80 samples ER+,35 ER-, analyzed separately fordistant tumor recurrence,then combined	Prognosis for distanttumor recurrence
Wound responsesignature	Chang *et al.* [18]	2004	512	50 fibroblast culturesfrom 10anatomic sites: response offibroblast to serum exposure	Prognosis formetastasis/survival
Lung metastasissignature	Minn *et al.* [19]	2005	54	Comparison of highly and weaklylung-metastatic cell populationsderived from the breast cancercell line MDA-MB-231	Prognosis for lungmetastasis
Brain metastasissignature	Bos *et al.* [20]	2009	243	Comparison of cell lines withdifferent metastatic potentialsderived from the breast cancercell lines MDA-MB-231 and CN34	Prognosis for brainmetastasis
Bone metastasissignature	Kang *et al.* [21]	2003	102	MDA-MB-231 breast cancer cell line+12 derivative subpopulations withdifferent metastatic potentials	Prognosis for bonemetastasis
Invasivenesssignature	Liu *et al.* [22]	2007	186	CD44+CD24−/low breast cancer cellswith high tumorgenic capacity vs. cellsof normal breast epithelium	Prognosis for overall/metastasis-free survival
General metastasissignature	Ramaswamy*et al.* [23]	2003	128	64 primary adenocarcinomas of diverseorigin (lung, breast, prostate,colorectal, uterus, ovary) vs. 12unmatched adenocarcinoma metastasis	Metastatic potential,clinical outcome
Meta genesignature	Györffy*et al.* [24]	2009	376	Meta-analysis of 20 published genesignatures on 7 breast cancermicroarray data sets (n = 1079)	Prognosis forrelapse-free survival
374 GeneSet/consensus genes	Lauss*et al.* [25]	2008	374	Meta-analysis of 44 published genesignatures on 8 breastcancer microarray datasets (n = 1067)	Prognosis forsurvival

The signatures could be divided in four subgroups. The commercially available gene signatures Mammaprint, Oncotype DX, MapQuant and Gene Search were dominated by the terms cell cycle and development (table 6). Also the two meta-analyses showed enrichment of genes involved in cell cycle (34% of the 50 top pathways each), as did the general metastasis signature (16%). The specific lung, brain and bone metastasis signatures showed a strong connection to the generic terms immune response and development, which represented about 30% and 20% of the enriched pathways, respectively, and they were lacking pathways associated with cell cycle. The wound response and invasiveness signature did not show any specific pattern.

### Comparison of the GWAS and the Gene Expression Signatures

**Figure 3:**
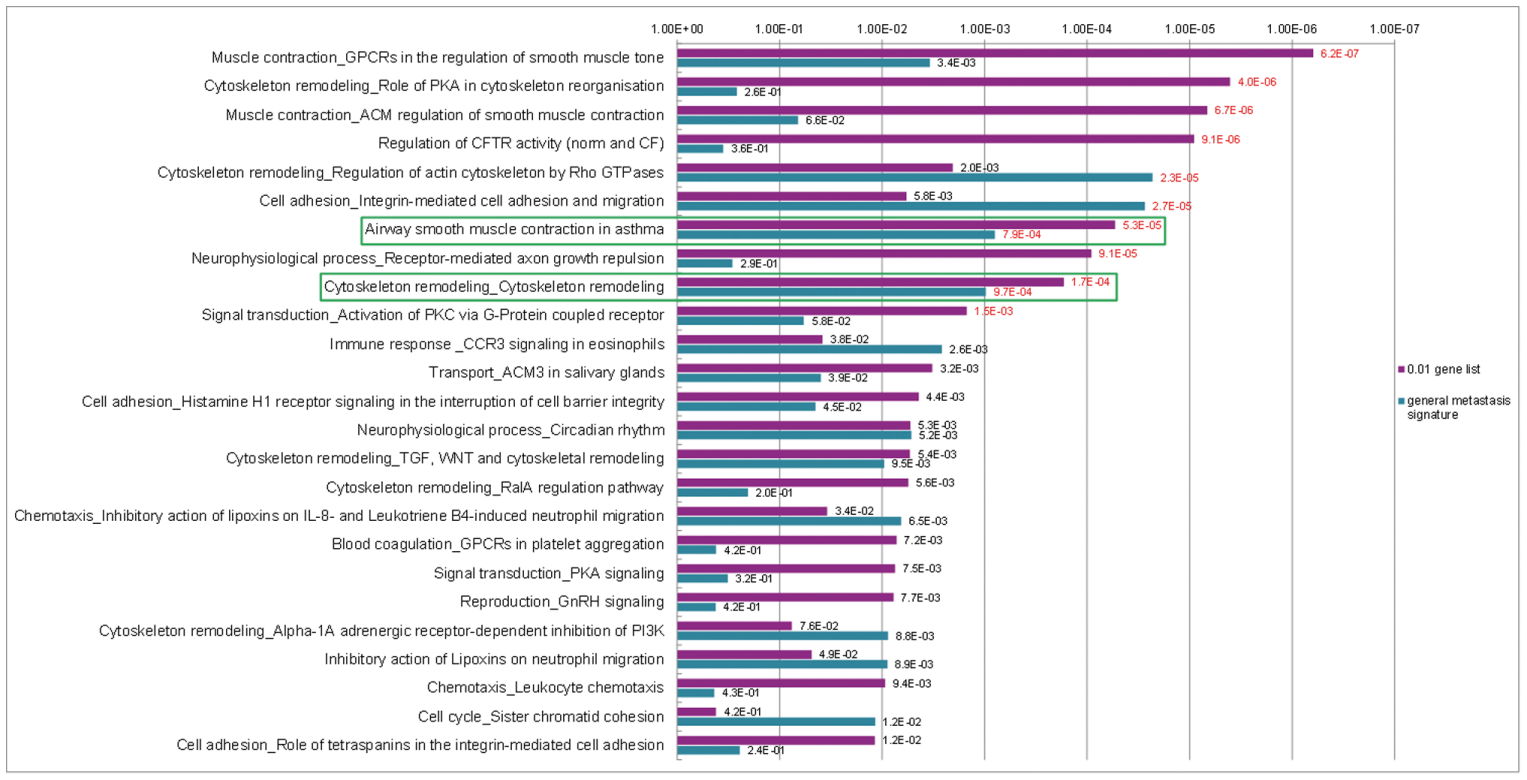
Top 25 GeneGO pathways enriched simultaneously by the 0.01 gene list and general metastasis signature; red numbers = significant at FDR of 0.05; green box = pathway significantly enriched by both gene lists; Pathway “Airway smooth muscle contraction in asthma” was placed at rank 7, pathway “Cytoskeleton remodeling_Cytoskeleton remodeling” was placed at rank 9.

In order to evaluate the similarities between the GWAS and the gene expression signatures, we analyzed the GWAS gene signature and every prognostic gene expression signature simultaneously with the MetaCore GeneGo pathway enrichment analysis tool to get a detailed view on their common pathways (figure 3, Figures S1–S11). In this analysis the two gene lists were investigated for overrepresented pathways and compared to each other. Only pathways enriched by both gene lists are displayed and ranked by their enrichment *p*-values. The pathways which were significantly enriched by both gene signatures at the same time were a rare event. In all simultaneous analyses, only three pathways passed the 0.05 FDR significance level in both analyzed gene lists contemporaneously. Two of them were enriched in the analysis of our GWAS gene list together with the general metastasis signature (figure 3). These were the “Airway smooth muscle contraction in asthma” pathway placed at rank 7 (P_0.01 gene list = _5.3×10^−5^; P_general metastasis signature = _7.9×10^−4^) and the “Cytoskelton remodeling_Cytoskelton remodeling” pathway (P_0.01 gene list = _1.7×10^−4^; P_general metastasis signature = _9.7×10^−4^) placed at rank 9.

**Figure 4:**
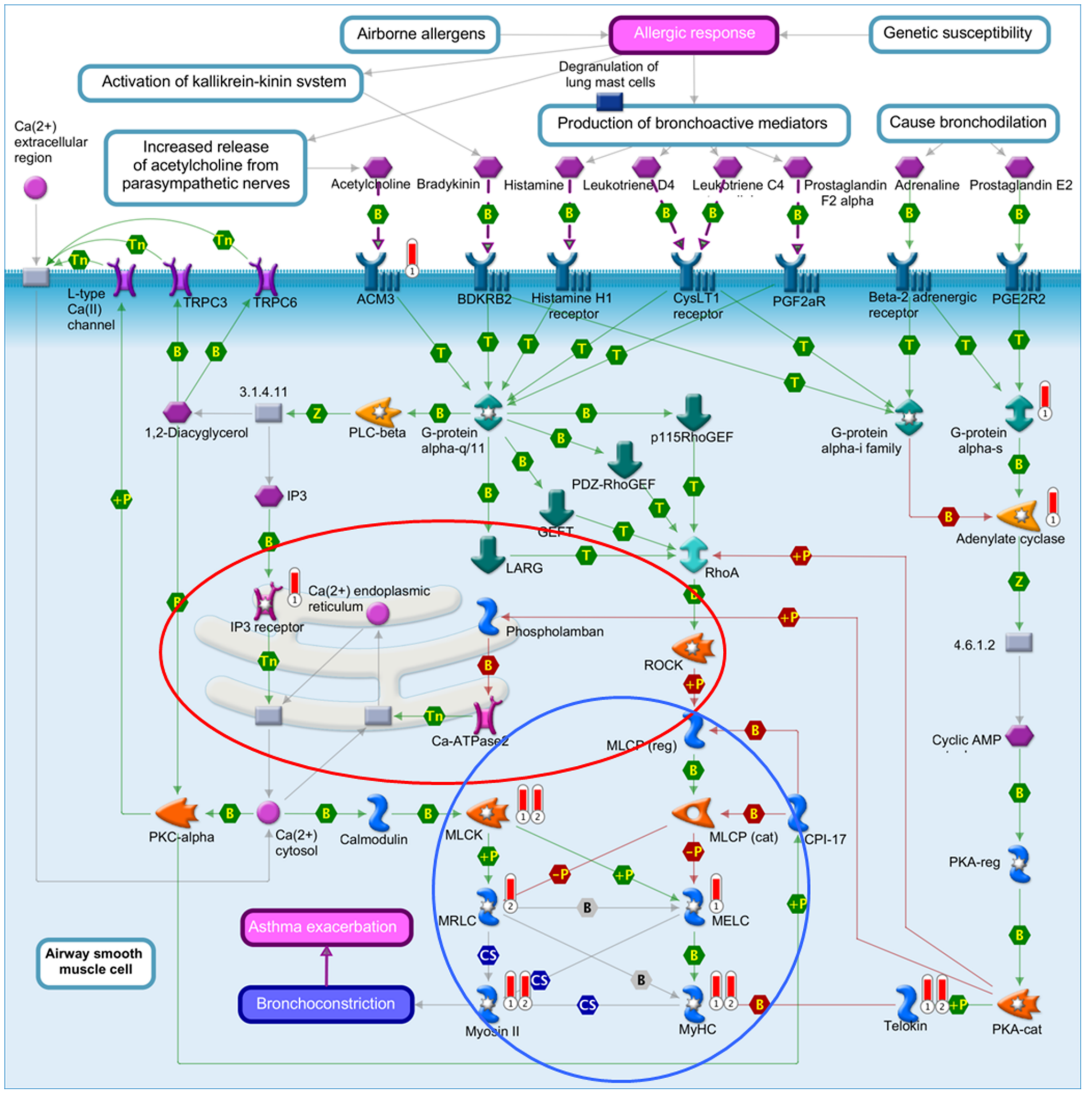
GeneGo pathway “Airway smooth muscle contraction in asthma”. Barometers: 1 = 0.01 gene list; 2 = general metastasis signature. red = Calcium signaling pathway, blue = Smooth muscle contraction/relaxation.

**Figure 5:**
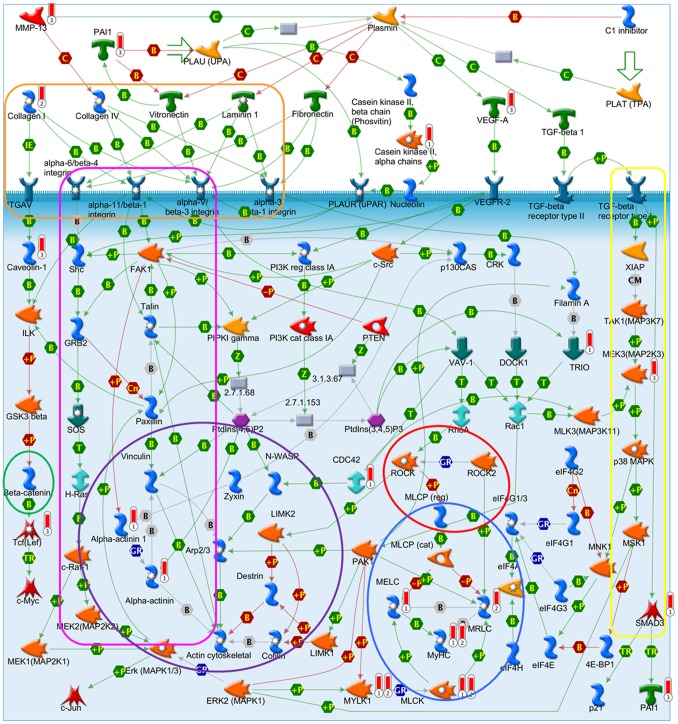
GeneGo pathway “Cytoskeleton remodeling”. Barometer: 1 = 0.01 gene list; 2 = general metastasis signature; 3 = brain metastasis signature. orange = ECM-receptor interaction, purple = Adherens junction pathway, red = Calcium signaling pathway, pink = Focal adhesion pathway, yellow = TGF-β signaling pathway, green = Wnt signaling pathway, blue = Smooth muscle contraction/relaxation.

“Airway smooth muscle contraction in asthma” (figure 4) is almost identical to the top pathway in this analysis, “Muscle contraction_GPCRs in the regulation of smooth muscle tone” (figure S12), showing a clear connection to calcium ion binding, with the Ca^2+^-ions containing endoplasmatic reticulum and the associated proteins as one central part of these pathways. Several proteins of these pathways can also be found in the GeneGo pathway “Cytoskeleton remodeling_Cytoskeleton remodeling” (figure 5). This pathway combines several sub-pathways, many of them involved in cell adhesion. These include the pathways “ECM-receptor interaction”, “focal adhesion” and “adherens junction”. Also links to the well-known cancer pathways “TGF-β signaling” and “Wnt signaling” are observed. “Cytoskeleton remodeling_Cytoskeleton remodeling” was also significantly enriched by the brain metastasis gene signature (P_brain metastasis signature = _2.5×10^−3^) and placed at rank 15 (figure S7).

**Figure 6:**
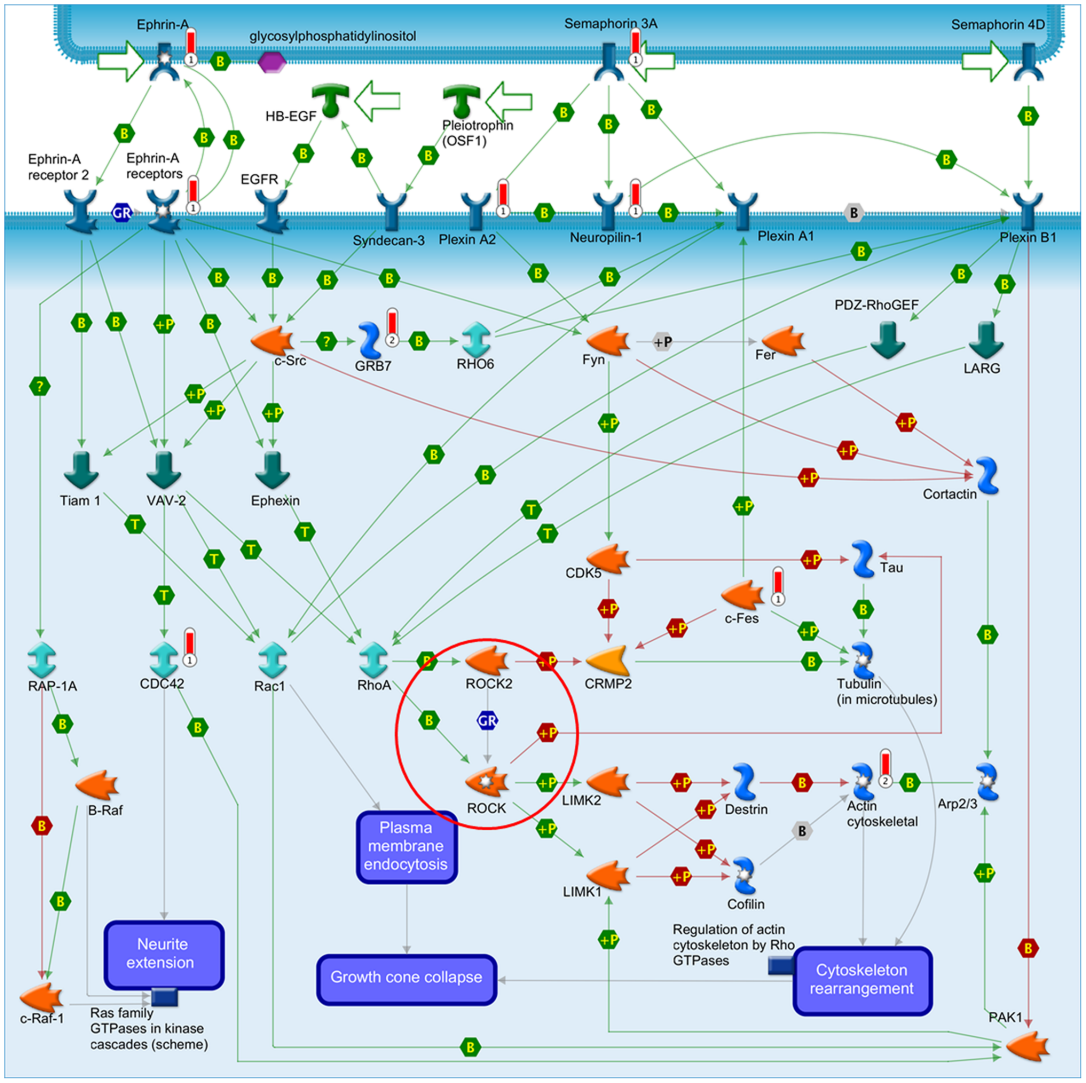
GeneGo pathway “Neurophysiological process_Receptor-mediated axon growth repulsion”. Barometers: 1 = 0.01 gene list; 2 = Oncotype DX. red = Calcium signaling pathway.

The third pathway significantly overrepresented by the 0.01 gene list and a gene expression signature in the simultaneous analyses was “Neurophysiological process_Receptor-mediated axon growth repulsion” (figure 6), which was significantly enriched in the analysis together with the Oncotype DX signature and placed at rank 4 (P_0.01 gene list = _9.1×10^−5^; P_Oncotype DX = _6.4×10^−3^) (figure S2). Also this pathway has a connection to the calcium signaling and cell adhesion pathway.

## Discussion

The aim of our study was to set data derived from a GWAS on breast cancer survival into a global context by using a systematic pathway enrichment analysis with the two independent databases GO and KEGG as basis. In this process, the GO database was searched for overrepresented terms on a higher level of abstraction. A more detailed and focused view was achieved by using the data of the KEGG. By this way we gained eleven consistently enriched categories, two more general GO terms and nine specific KEGG pathways, which may have an influence on BC survival. A gene overlap of up to 87% between six of these categories revealed a strong connection to cell adhesion and included the KEGG pathways “adherens junction”, “axon guidance” “ECM-receptor interaction”, “focal adhesion”, and “small cell lung cancer” and the GO term “cell adhesion”. The second category with a high proportion of overlapping genes involved in calcium ion binding included the GO term “calcium ion binding” and the KEGG pathways “arrythmogenic right ventricular cardiomyopathy”, “calcium signaling”, “dilated cardiomyopathy” and “O-glycan biosynthesis”. There was also an overlap of 20–30% between the two overarching categories, which emphasizes the interplay of cellular adhesion processes and calcium signaling as an important process in breast cancer survival.

In the second part of our study we compared the pathway enrichment results of our GWAS data to those of twelve prognostic gene expression signatures. Simultaneously conducted pathway enrichment analyses with each of the expression signatures revealed that the “Airway smooth muscle contraction in asthma”, the “Cytoskeleton remodeling” and the “Neurophysiological process_Receptor-mediated axon growth repulsion” pathways were the only ones which were significantly overrepresented by both the GWAS and a gene expression signature. The gene expression signatures involved were the general metastasis signature with two simultaneously enriched pathways and the specific brain metastasis signature and Oncotype DX each with one simultaneously enriched pathway, respectively. The general metastasis signature was derived from a comparison of gene expression data of adenocarcinomas of diverse origin (lung, breast, prostate, colorectal, uterus, ovary) with the corresponding metastases leading to 128 genes that distinguished best between primary tumors and metastases [23]. The brain metastasis signature is based on a genome-wide expression analysis of two BC cell lines and their highly brain metastatic cell derivates [20]. The comparison of the gene expression profiles led to 243 differentially expressed genes, which were used as a brain metastasis signature in our study. The Oncotype DX signature was generated by a hypothesis driven search of the literature and databases for candidate cancer genes, which were tested for their correlation with disease recurrence in three independent breast cancer studies [15]. The sixteen best performing genes and five reference genes were used to calculate a recurrence score. These three gene expression signatures are based on genes which are already known for their involvement in cancer (Oncotype DX) or which are associated with the metastasis forming process (general and brain metastasis signature). Together, the three simultaneously enriched pathways picture well a possible interaction of the cell adhesion pathways with the calcium signaling pathway in the metastatic process and the patients’ survival probabilities (figures 4–6).

Calcium signaling and cell adhesion interact in various ways with each other and play an important role in metastasis, which involves detachment from the solid primary tumor, migration and invasion in a foreign tissue [26]. For example, E-cadherin as a key cell-to-cell adhesion molecule, essentially requires Ca^2+^-ions to form homophilic interactions between two neighboring cells in adherens junction [27]. Its down-regulation or inactivation in carcinomas has been reported to result in reduced cell adhesion [28], [29] making it as a major suppressor of metastasis. Also focal adhesions, as the main linkage point between the cells and the extra cellular matrix (ECM), are influenced by calcium. Focal adhesion turnover, which determines the efficiency of cell migration, is regulated by calcium signaling. An important component in this process is focal adhesion kinase (FAK), which is a contact point for diverse extracellular stimuli, including Ca^2+^-concentration. FAK coordinates signals between integrins, the attachment molecules to the ECM, and growth factor receptors and promotes cell migration [30]. These examples point to the regulation of the metastasis formation either directly through mutations in the involved adhesion molecules or indirectly through impaired “calcium signaling”.

Metastases are the leading cause of death of cancer patients and therefore strongly connected to patients’ survival. This was also reflected in our study population: short-time survivors tended to have tumors with higher stage than long-time survivors. As our data is based on a GWAS on BC survival comparing women with short-time survival to those with long-time survival, the results of our pathway enrichment analyses reflect the impact of the invasive tumor phenotype on the survival of a patient. Moreover, the comparison analyses with the pathway enrichment results of commonly used prognostic gene expression signatures support our conclusion.

Although pathway enrichment tools are able to put the GWAS data into a global context, there are some points which need to be considered [12], [31]. Large genes with more SNPs are more likely to contain associated SNPs by chance alone than small genes. To avoid this bias, we annotated the SNPs to a gene both by excluding and including a 20 kb up- and downstream sequence of the gene. Only the best SNP (i.e. the one with the lowest *p*-value) per gene was included in the analysis. The 20 kb limit was applied because the average length of haplotype blocks in the CEU population ranges between 5.9 kb (calculation method based on the four gamete test) and 16.3 kb (calculation method based on a composite of local D′ values) [32]. The pathway enrichment tools themselves also suffer from some limitations, which we experienced in our study. Even though the conditions were identical in all analyses, the different tools showed large variability in the number of overrepresented categories and their corresponding *p*-values [33]. The reasons for this variation include the source and the version of the annotation files, the annotation level used by the tool, the statistical model applied for the enrichment analysis, the correction for multiple testing, and the background gene set, which is used to calculate the *p*-values for the overrepresented pathways [34]. One way to avoid the problem of inconsistent results obtained by different tools is to use several tools and to compare the results with each other. In our study, we analyzed four gene lists derived from the GWAS on BC survival with six tools and compared the results to detect true, consistently enriched categories.

In conclusion, our pathway enrichment analysis of the high-throughput data from a GWAS on BC survival revealed an influence of cell adhesion and calcium signaling on BC patients’ survival. This was also confirmed by our comparison to the enrichment analyses of twelve prognostic gene expression signatures. The known high impact of metastasis on a patients’ survival is supported by our genetic data, which also highlights the influence of changes in cell adhesion and calcium signaling in the metastatic process. Therefore, a further investigation of the identified pathways and the defined mechanisms of metastasis is a promising target to get classifiers for the patients’ survival.

## Supporting Information

Figure S1 **Top 25 GeneGO pathways enriched simultaneously by the 0.01 gene list and Mammaprint.** red numbers = significant at FDR of 0.05.
(TIF)

Figure S2 **Top 25 GeneGO pathways enriched simultaneously by the 0.01 gene list and Oncotype DX.** red numbers = significant at FDR of 0.05; green box = “Neurophysiological process_Receptor mediated axon growth repulsion” pathway significantly enriched by both gene lists at rank 4.
(TIF)

Figure S3 **Top 25 GeneGO pathways enriched simultaneously by the 0.01 gene list and MapQuant.** red numbers = significant at FDR of 0.05.
(TIF)

Figure S4 **Top 25 GeneGO pathways enriched simultaneously by the 0.01 gene list and Gene Search.** red numbers = significant at FDR of 0.05.
(TIF)

Figure S5 **Top 25 GeneGO pathways enriched simultaneously by the 0.01 gene list and wound response signature.** red numbers = significant at FDR of 0.05; green box = pathway significantly enriched by both gene lists.
(TIF)

Figure S6 **Top 25 GeneGO pathways enriched simultaneously by the 0.01 gene list and the lung metastasis signature.** red numbers = significant at FDR of 0.05.
(TIF)

Figure S7 **Top 25 GeneGO pathways enriched simultaneously by the 0.01 gene list and the brain metastasis signature.** red numbers = significant at FDR of 0.05; green box = “Cytoskeleton remodeling_ Cytoskeleton remodeling” pathway, significantly enriched by both gene lists at rank 15.
(TIF)

Figure S8 **Top 25 GeneGO pathways enriched simultaneously by the 0.01 gene list and the bone metastasis signature, red numbers = significant at FDR of 0.05.**
(TIF)

Figure S9 **Top 25 GeneGO pathways enriched simultaneously by the 0.01 gene list and the invasiveness signature. red numbers = significant at FDR of 0.05.**
(TIF)

Figure S10 **Top 25 GeneGO pathways enriched simultaneously by the 0.01 gene list and the meta gene signature. red numbers = significant at FDR of 0.05.**
(TIF)

Figure S11 **Top 25 GeneGO pathways enriched simultaneously by the 0.01 gene list and the consensus gene signature.** red numbers = significant at FDR of 0.05.
(TIF)

Figure S12 **GeneGo pathway “Muscle contraction_GPCRs in the regulation of smooth muscle tone”.** Barometers: 1 = 0.01 gene list; 2 = general metastasis signature; red = Calcium signaling pathway.
(TIF)

Table S1 **Detailed characteristics of the whole study population.**
(DOCX)

Table S2 **General characteristics of sub-populations used in the GWAS.**
(DOCX)

Table S3 **Used pathway enrichment tools and their features.**
(DOCX)

Table S4 **Top 50 GeneGo pathways enriched by the 0.01 gene list.**
(DOCX)

## References

[1] Jemal A, Bray F, Center MM, Ferlay J, Ward E, et al (2011) Global cancer statistics. CA Cancer J Clin 61: 69–90. 21296855 10.3322/caac.20107

[2] Hemminki K, Ji J, Forsti A, Sundquist J, Lenner P (2008) Survival in breast cancer is familial. Breast Cancer Res Treat 110: 177–182. 17674192 10.1007/s10549-007-9692-7

[3] Shu XO, Long J, Lu W, Li C, Chen WY, et al (2012) Novel genetic markers of breast cancer survival identified by a genome-wide association study. Cancer Res 72: 1182–1189. 22232737 10.1158/0008-5472.CAN-11-2561 PMC3294129

[4] Azzato EM, Pharoah PD, Harrington P, Easton DF, Greenberg D, et al (2010) A genome-wide association study of prognosis in breast cancer. Cancer Epidemiol Biomarkers Prev 19: 1140–1143. 20332263 10.1158/1055-9965.EPI-10-0085 PMC2852476

[5] Azzato EM, Tyrer J, Fasching PA, Beckmann MW, Ekici AB, et al (2010) Association between a germline OCA2 polymorphism at chromosome 15q13.1 and estrogen receptor-negative breast cancer survival. J Natl Cancer Inst 102: 650–662. 20308648 10.1093/jnci/djq057 PMC2864289

[6] Huang da W, Sherman BT, Lempicki RA (2008) Bioinformatics enrichment tools: paths toward the comprehensive functional analysis of large gene lists. Nucleic Acids Res 37: 1–13. 19033363 10.1093/nar/gkn923 PMC2615629

[7] Kaaks R, Lundin E, Rinaldi S, Manjer J, Biessy C, et al (2002) Prospective study of IGF-I, IGF-binding proteins, and breast cancer risk, in northern and southern Sweden. Cancer Causes Control 13: 307–316. 12074500 10.1023/a:1015270324325

[8] Manjer J, Carlsson S, Elmstahl S, Gullberg B, Janzon L, et al (2001) The Malmo Diet and Cancer Study: representativity, cancer incidence and mortality in participants and non-participants. Eur J Cancer Prev 10: 489–499. 11916347 10.1097/00008469-200112000-00003

[9] Pukkala E, Andersen A, Berglund G, Gislefoss R, Gudnason V, et al (2007) Nordic biological specimen banks as basis for studies of cancer causes and control–more than 2 million sample donors, 25 million person years and 100,000 prospective cancers. Acta Oncol 46: 286–307. 17450464 10.1080/02841860701203545

[10] Tryggvadottir L, Sigvaldason H, Olafsdottir GH, Jonasson JG, Jonsson T, et al (2006) Population-based study of changing breast cancer risk in Icelandic BRCA2 mutation carriers, 1920–2000. J Natl Cancer Inst 98: 116–122. 16418514 10.1093/jnci/djj012

[11] Lascorz J, Chen B, Hemminki K, Forsti A (2011) Consensus pathways implicated in prognosis of colorectal cancer identified through systematic enrichment analysis of gene expression profiling studies. PLoS One 6: e18867. 21541025 10.1371/journal.pone.0018867 PMC3081819

[12] Elbers CC, van Eijk KR, Franke L, Mulder F, van der Schouw YT, et al (2009) Using genome-wide pathway analysis to unravel the etiology of complex diseases. Genet Epidemiol 33: 419–431. 19235186 10.1002/gepi.20395

[13] Holmans P, Green EK, Pahwa JS, Ferreira MA, Purcell SM, et al (2009) Gene ontology analysis of GWA study data sets provides insights into the biology of bipolar disorder. Am J Hum Genet 85: 13–24. 19539887 10.1016/j.ajhg.2009.05.011 PMC2706963

[14] van ‘t Veer LJ, Dai H, van de Vijver MJ, He YD, Hart AA, et al (2002) Gene expression profiling predicts clinical outcome of breast cancer. Nature 415: 530–536. 11823860 10.1038/415530a

[15] Paik S, Shak S, Tang G, Kim C, Baker J, et al (2004) A multigene assay to predict recurrence of tamoxifen-treated, node-negative breast cancer. N Engl J Med 351: 2817–2826. 15591335 10.1056/NEJMoa041588

[16] Sotiriou C, Wirapati P, Loi S, Harris A, Fox S, et al (2006) Gene expression profiling in breast cancer: understanding the molecular basis of histologic grade to improve prognosis. J Natl Cancer Inst 98: 262–272. 16478745 10.1093/jnci/djj052

[17] Wang Y, Klijn JG, Zhang Y, Sieuwerts AM, Look MP, et al (2005) Gene-expression profiles to predict distant metastasis of lymph-node-negative primary breast cancer. Lancet 365: 671–679. 15721472 10.1016/S0140-6736(05)17947-1

[18] Chang HY, Sneddon JB, Alizadeh AA, Sood R, West RB, et al (2004) Gene expression signature of fibroblast serum response predicts human cancer progression: similarities between tumors and wounds. PLoS Biol 2: E7. 14737219 10.1371/journal.pbio.0020007 PMC314300

[19] Minn AJ, Gupta GP, Siegel PM, Bos PD, Shu W, et al (2005) Genes that mediate breast cancer metastasis to lung. Nature 436: 518–524. 16049480 10.1038/nature03799 PMC1283098

[20] Bos PD, Zhang XH, Nadal C, Shu W, Gomis RR, et al (2009) Genes that mediate breast cancer metastasis to the brain. Nature 459: 1005–1009. 19421193 10.1038/nature08021 PMC2698953

[21] Kang Y, Siegel PM, Shu W, Drobnjak M, Kakonen SM, et al (2003) A multigenic program mediating breast cancer metastasis to bone. Cancer Cell 3: 537–549. 12842083 10.1016/s1535-6108(03)00132-6

[22] Liu R, Wang X, Chen GY, Dalerba P, Gurney A, et al (2007) The prognostic role of a gene signature from tumorigenic breast-cancer cells. N Engl J Med 356: 217–226. 17229949 10.1056/NEJMoa063994

[23] Ramaswamy S, Ross KN, Lander ES, Golub TR (2003) A molecular signature of metastasis in primary solid tumors. Nat Genet 33: 49–54. 12469122 10.1038/ng1060

[24] Gyorffy B, Schafer R (2009) Meta-analysis of gene expression profiles related to relapse-free survival in 1,079 breast cancer patients. Breast Cancer Res Treat 118: 433–441. 19052860 10.1007/s10549-008-0242-8

[25] Lauss M, Kriegner A, Vierlinger K, Visne I, Yildiz A, et al (2008) Consensus genes of the literature to predict breast cancer recurrence. Breast Cancer Res Treat 110: 235–244. 17899371 10.1007/s10549-007-9716-3

[26] Talmadge JE, Fidler IJ (2010) AACR centennial series: the biology of cancer metastasis: historical perspective. Cancer Res 70: 5649–5669. 20610625 10.1158/0008-5472.CAN-10-1040 PMC4037932

[27] Baum B, Georgiou M (2011) Dynamics of adherens junctions in epithelial establishment, maintenance, and remodeling. J Cell Biol 192: 907–917. 21422226 10.1083/jcb.201009141 PMC3063136

[28] Berx G, van Roy F (2009) Involvement of members of the cadherin superfamily in cancer. Cold Spring Harb Perspect Biol 1: a003129. 20457567 10.1101/cshperspect.a003129 PMC2882122

[29] Cavallaro U, Christofori G (2004) Cell adhesion and signalling by cadherins and Ig-CAMs in cancer. Nat Rev Cancer 4: 118–132. 14964308 10.1038/nrc1276

[30] Li S, Hua ZC (2008) FAK expression regulation and therapeutic potential. Adv Cancer Res 101: 45–61. 19055942 10.1016/S0065-230X(08)00403-X

[31] Wang K, Li M, Hakonarson H (2010) Analysing biological pathways in genome-wide association studies. Nat Rev Genet 11: 843–854. 21085203 10.1038/nrg2884

[32] International HapMap C (2005) A haplotype map of the human genome. Nature 437: 1299–1320. 16255080 10.1038/nature04226 PMC1880871

[33] Rhee SY, Wood V, Dolinski K, Draghici S (2008) Use and misuse of the gene ontology annotations. Nat Rev Genet 9: 509–515. 18475267 10.1038/nrg2363

[34] Khatri P, Draghici S (2005) Ontological analysis of gene expression data: current tools, limitations, and open problems. Bioinformatics 21: 3587–3595. 15994189 10.1093/bioinformatics/bti565 PMC2435250

