# Supplementary figures and images for: Systematic Pathway Enrichment Analysis of a Genome-Wide Association Study on Breast Cancer Survival Reveals an Influence of Genes Involved in Cell Adhesion and Calcium Signaling on the Patients’ Clinical Outcome
Source: PLoS One. 2014 Jun 2;9(6):e98229. doi: 10.1371/journal.pone.0098229 (PMC4041745; doi:10.1371/journal.pone.0098229)

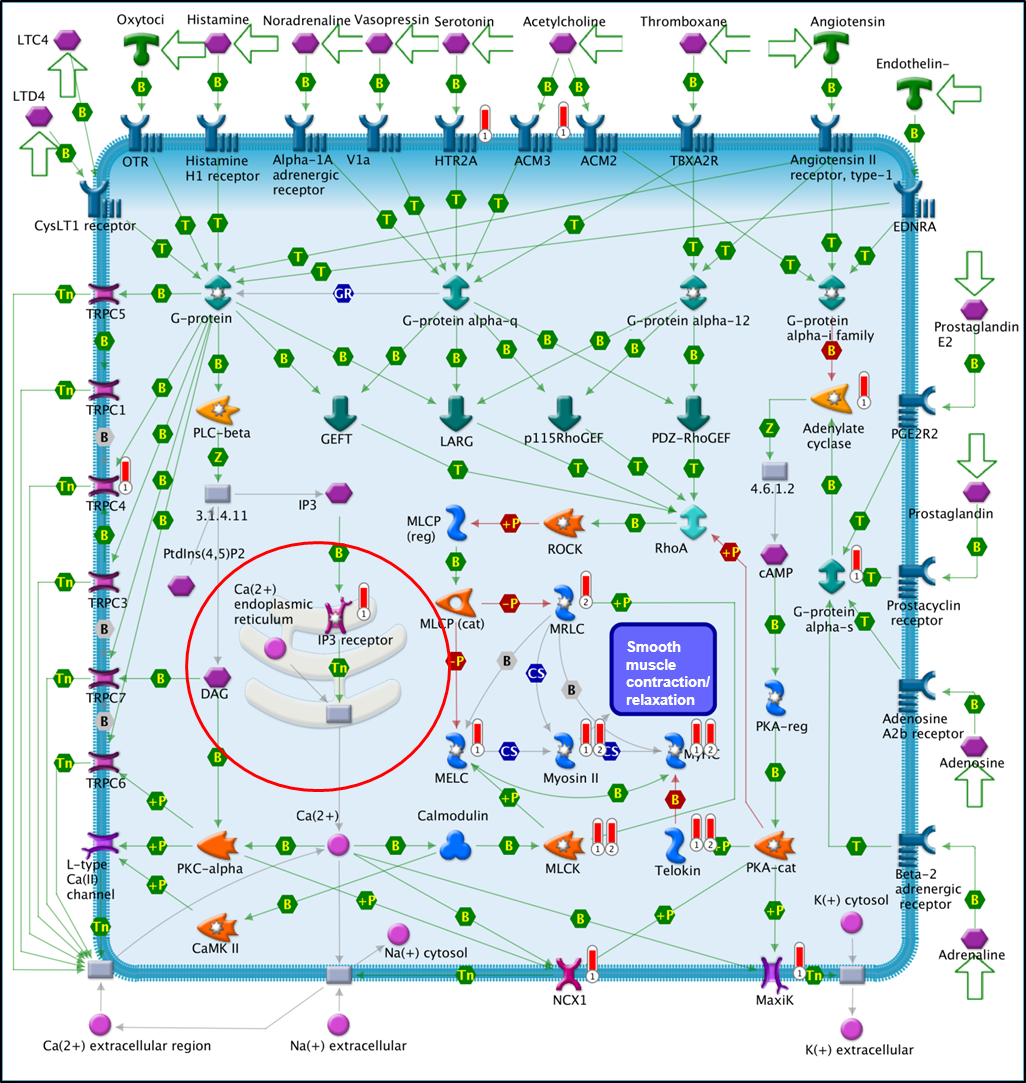

Supplement: Figure S12 — GeneGo pathway “Muscle contraction_GPCRs in the regulation of smooth muscle tone”. Barometers: 1 = 0.01 gene list; 2 = general metastasis signature; red = Calcium signaling pathway. (TIF) [file pone.0098229.s012.tif]
