# Supplementary material for: Systematic Pathway Enrichment Analysis of a Genome-Wide Association Study on Breast Cancer Survival Reveals an Influence of Genes Involved in Cell Adhesion and Calcium Signaling on the Patients’ Clinical Outcome
Source: PLoS One. 2014 Jun 2;9(6):e98229. doi: 10.1371/journal.pone.0098229 (PMC4041745; doi:10.1371/journal.pone.0098229)
Supplement: Table S1 — Detailed characteristics of the whole study population. (DOCX) [file pone.0098229.s013.docx]

Table S1: Detailed characteristics of the whole study population

|  | **All populations** | | | |
| --- | --- | --- | --- | --- |
|  | **cases*** | | **controls**** | |
| **age±std(years)** | 59.7±12.9 | | 58.4±11.3 | |
|  | No. | % | No. | % |
| total | 369 | 100.0 | 369 | 100.0 |
|  |  |  |  |  |
| **stage** | | | | |
| 0 | 0 | 0.0 | 3 | 0.8 |
| 1 | 44 | 12.2 | 212 | 58.1 |
| 2 | 186 | 51.4 | 130 | 35.6 |
| 3 | 72 | 19.9 | 19 | 5.2 |
| 4 | 60 | 16.6 | 1 | 0.3 |
| total | 362 | 98.1 | 365 | 98.9 |
| missing | 7 | 1.9 | 4 | 1.9 |
|  |  |  |  |  |
| **size** | | | | |
| ≤2cm | 82 | 31.3 | 239 | 72.6 |
| >2cm | 180 | 68.7 | 90 | 27.4 |
| total | 262 | 71.0 | 329 | 89.2 |
| missing | 107 | 29.0 | 40 | 10.8 |
|  |  |  |  |  |
| **Lymph node status(N)** | | | | |
| negative | 71 | 25.7 | 215 | 71.0 |
| positive | 205 | 74.3 | 88 | 29.0 |
| total | 276 | 74.8 | 303 | 82.1 |
| missing | 93 | 25.2 | 66 | 17.9 |
|  |  |  |  |  |
| **grade** | | | | |
| 1 | 6 | 2.5 | 52 | 25.2 |
| 2 | 79 | 32.8 | 97 | 47.1 |
| 3 | 156 | 64.7 | 57 | 27.7 |
| total | 241 | 65.3 | 206 | 55.8 |
| missing | 128 | 34.7 | 163 | 44.2 |
|  |  |  |  |  |
| **Estrogen receptor status** | | | | |
| positive | 157 | 51.8 | 162 | 74.3 |
| negative | 146 | 48.2 | 56 | 25.7 |
| total | 303 | 82.1 | 218 | 59.1 |
| missing | 66 | 17.9 | 151 | 40.9 |
|  |  |  |  |  |
| **Progesterone receptor status** | | | | |
| positive | 105 | 36.8 | 124 | 59.6 |
| negative | 180 | 63.2 | 84 | 40.4 |
| total | 285 | 77.2 | 208 | 56.4 |
| missing | 84 | 22.8 | 161 | 43.6 |
|  |  |  |  |  |
| **ER/PR status** | | | | |
| +/+ | 83 | 30.3 | 93 | 49.7 |
| +/- | 51 | 18.6 | 38 | 20.3 |
| -/+ | 19 | 6.9 | 15 | 8.0 |
| -/- | 121 | 44.2 | 41 | 21.9 |
| total | 274 | 74.3 | 187 | 50.7 |
| missing | 95 | 25.7 | 182 | 49.3 |
| *BCpatientswithshort-timesurvival(<6yearsafterBCdiagnosis) | | | | |
| **BCpatientswithlong-termsurvivaol(≥11yearsafterBCdiagnosis) | | | | |
