# Supplementary material for: Systematic Pathway Enrichment Analysis of a Genome-Wide Association Study on Breast Cancer Survival Reveals an Influence of Genes Involved in Cell Adhesion and Calcium Signaling on the Patients’ Clinical Outcome
Source: PLoS One. 2014 Jun 2;9(6):e98229. doi: 10.1371/journal.pone.0098229 (PMC4041745; doi:10.1371/journal.pone.0098229)
Supplement: Table S2 — General characteristics of sub-populations used in the GWAS. (DOCX) [file pone.0098229.s014.docx]

Table S2: General characteristics of sub-populations used in the GWAS

|  | **All populations** | | **Umeå** | | **Malmö** | | **Iceland** | | **Germany** | |  |
| --- | --- | --- | --- | --- | --- | --- | --- | --- | --- | --- | --- |
|  | **cases*** | **controls**** | **cases*** | **controls**** | **cases*** | **controls**** | **cases*** | **controls**** | **cases*** | **controls**** |  |
| **No.** | 369 | 369 | 96 | 96 | 44 | 44 | 147 | 147 | 82 | 82 |  |
| **age ± std (years)** | 59.7 ± 12.9 | 58.4 ± 11.3 | 57.9 ± 10.8 | 56.0 ± 9.2 | 62.1 ±7.9 | 60.1 ±6.4 | 60.5 ± 15.4 | 59.4 ±13.5 | 59.2 ± 11.7 | 58.2 ± 8.0 |  |
| **diagnosis period (calendar years)** |  |  | 1988-2005 | | 1991-2005 | | 1985-2005 | | 1993-2005 | |  |
| *BC patients with short-time survival (< 6 years after BC diagnosis) | | | | | | | | | | | |
| ** BC patients with long-time survival (≥ 11 years after BC diagnosis) | | | | | | | | | | | |
