# Supplementary material for: Systematic Pathway Enrichment Analysis of a Genome-Wide Association Study on Breast Cancer Survival Reveals an Influence of Genes Involved in Cell Adhesion and Calcium Signaling on the Patients’ Clinical Outcome
Source: PLoS One. 2014 Jun 2;9(6):e98229. doi: 10.1371/journal.pone.0098229 (PMC4041745; doi:10.1371/journal.pone.0098229)
Supplement: Table S3 — Used pathway enrichment tools and their features. (DOCX) [file pone.0098229.s015.docx]

Table S3: Used pathway enrichment tools and their features

| **Tool name** | **Used Databases** | **Key statistical method** | **Multiple testing correction method** |
| --- | --- | --- | --- |
| ConsensusPathDB | BP/MF/KEGG | Hypergeometric | FDR |
| DAVID | BP/MF/KEGG | Fisher‘s exact | Benjamini |
| FatiGO | BP/MF/KEGG | Fisher‘s exact | BH |
| GATHER | BP/MF/KEGG | Bayes factor | FDR |
| GeneCodis | BP/MF/KEGG | Hypergeometric | FDR |
| WebGestalt | BP/ MF/KEGG | Hypergeometric | BH |
| BH = Benjamini-Hochberg; BP = Biological Process; FDR = False Discovery Rate; MF = Molecular Function | | | |
