# Supplementary material for: Systematic Pathway Enrichment Analysis of a Genome-Wide Association Study on Breast Cancer Survival Reveals an Influence of Genes Involved in Cell Adhesion and Calcium Signaling on the Patients’ Clinical Outcome
Source: PLoS One. 2014 Jun 2;9(6):e98229. doi: 10.1371/journal.pone.0098229 (PMC4041745; doi:10.1371/journal.pone.0098229)
Supplement: Table S4 — Top 50 GeneGo pathways enriched by the 0.01 gene list. (DOCX) [file pone.0098229.s016.docx]

Table S4: Top 50 GeneGo pathways enriched by the 0.01 gene list

| **Rank** | **GeneGo generic term** | **Defined GeneGo pathway** | ***p-*value** |
| --- | --- | --- | --- |
| 1 | Muscle contraction | GPCRs in the regulation of smooth muscle tone | 6.0E-07 |
| 2 | Cytoskeleton remodeling | Role of PKA in cytoskeleton reorganisation | 3.9E-06 |
| 3 | Muscle contraction | ACM regulation of smooth muscle contraction | 6.5E-06 |
| 4 |  | Regulation of CFTR activity (norm and CF) | 8.8E-06 |
| 5 |  | O-glycan biosynthesis | 1.8E-05 |
| 6 |  | O-glycan biosynthesis / Human version | 1.8E-05 |
| 7 | Development | Beta-adrenergic receptors signaling via cAMP | 3.0E-05 |
| 8 | Neurophysiological process | Netrin-1 in regulation of axon guidance | 4.8E-05 |
| 9 | Neurophysiological process | Receptor-mediated axon growth repulsion | 8.9E-05 |
| 10 | Cytoskeleton remodeling | Cytoskeleton remodeling | 1.6E-04 |
| 11 | Regulation of lipid metabolism | Stimulation of Arachidonic acid production by ACM receptors | 3.2E-04 |
| 12 | Neurophysiological process | ACM regulation of nerve impulse | 7.6E-04 |
| 13 | Cell adhesion | Alpha-4 integrins in cell migration and adhesion | 1.2E-03 |
| 14 | Signal transduction | Activation of PKC via G-Protein coupled receptor | 1.5E-03 |
| 15 | Cytoskeleton remodeling | Regulation of actin cytoskeleton by Rho GTPases | 2.0E-03 |
| 16 | Signal transduction | cAMP signaling | 2.0E-03 |
| 17 | Neurophysiological process | Delta-type opioid receptor in the nervous system | 2.6E-03 |
| 18 | Apoptosis and survival | BAD phosphorylation | 3.2E-03 |
| 19 | Transport | ACM3 in salivary glands | 3.2E-03 |
| 20 |  | cAMP/ Ca(2+)-dependent Insulin secretion | 3.5E-03 |
| 21 | Cell adhesion | Histamine H1 receptor signaling in the interruption of cell barrier integrity | 4.3E-03 |
| 22 | Signal transduction | Calcium signaling | 4.3E-03 |
| 23 | G-protein signaling | Regulation of cAMP levels by ACM | 4.3E-03 |
| 24 | Immune response | MIF - the neuroendocrine-macrophage connector | 4.8E-03 |
| 25 | Neurophysiological process | Circadian rhythm | 5.2E-03 |
| 26 | Transport | Alpha-2 adrenergic receptor regulation of ion channels | 5.2E-03 |
| 27 | Cytoskeleton remodeling | TGF, WNT and cytoskeletal remodeling | 5.2E-03 |
| 28 | Cytoskeleton remodeling | RalA regulation pathway | 5.5E-03 |
| 29 | Cell adhesion | Integrin-mediated cell adhesion and migration | 5.7E-03 |
| 30 |  | Cholesterol and Sphingolipids transport / Distribution to the intracellular membrane compartments (normal and CF) | 6.2E-03 |
| 31 | Development | A2B receptor: action via G-protein alpha s | 6.8E-03 |
| 32 | Blood coagulation | GPCRs in platelet aggregation | 7.1E-03 |
| 33 | Signal transduction | PKA signaling | 7.4E-03 |
| 34 | Reproduction | GnRH signaling | 7.6E-03 |
| 35 | G-protein signaling | Regulation of CDC42 activity | 7.7E-03 |
| 36 | Cytoskeleton remodeling | Thyroliberin in cytoskeleton remodeling | 7.7E-03 |
| 37 | Muscle contraction | Role of kappa-type opioid receptor in heart | 7.7E-03 |
| 38 | G-protein signaling | RhoA regulation pathway | 8.6E-03 |
| 39 | Development | Endothelin-1/EDNRA signaling | 8.7E-03 |
| 40 | Chemotaxis | Leukocyte chemotaxis | 9.2E-03 |
| 41 | Development | Keratinocyte differentiation | 1.1E-02 |
| 42 | Cell adhesion | Role of tetraspanins in the integrin-mediated cell adhesion | 1.2E-02 |
| 43 | Transcription | ChREBP regulation pathway | 1.3E-02 |
| 44 | Development | VEGF-family signaling | 1.7E-02 |
| 45 | Cell adhesion | Role of CDK5 in cell adhesion | 1.9E-02 |
| 46 | Cell adhesion | Endothelial cell contacts by non-junctional mechanisms | 1.9E-02 |
| 47 | Development | GDNF signaling | 1.9E-02 |
| 48 | Neurophysiological process | Melatonin signaling | 1.9E-02 |
| 49 | Development | A2A receptor signaling | 1.9E-02 |
| 50 | Cardiac Hypertrophy | NF-AT signaling in Cardiac Hypertrophy | 2.0E-02 |
